# Multimorbidity in elderly patients with or without T2DM: A real-world cross-sectional analysis based on primary care and hospitalisation data

**DOI:** 10.7189/jogh.14.04263

**Published:** 2024-12-20

**Authors:** Yang Li, Shasha Geng, Huixiao Yuan, Jianli Ge, Qingqing Li, Xin Chen, Yingqian Zhu, Yue Liu, Xiaotong Guo, Xiaoli Wang, Hua Jiang

**Affiliations:** 1 Department of General Practice, Shanghai East Hospital, Tongji University School of Medicine, Shanghai, China; 2 Department of Geriatrics, Shanghai East Hospital, Tongji University School of Medicine, Shanghai, China; 3 Pudong Institute for Health Development, Shanghai, China

## Abstract

**Background:**

Shanghai's high level of ageing has given rise to a considerable number of elderly patients with type 2 diabetes mellitus (T2DM) who are confronted with the challenge of managing multimorbidity. We aimed to determine the prevalence of multimorbidity in elderly T2DM patients in a representative Pudong New Area community and critically evaluate current guidelines' inclusiveness in addressing major comorbidities.

**Methods:**

Through the Shanghai Health Cloud platform, we extracted medical records of residents in the Huamu community (Pudong New Area, Shanghai) to screen elderly patients with at least three outpatient visits or one hospitalisation per year between 2019 and 2022. According to International Classification of Disease, 10th edition codes and personal identification number, we identified the status of T2DM and 12 other common chronic diseases, matched T2DM patients and non-T2DM patients 1:1 by age and gender, and then calculated the prevalence of multimorbidity status and annual prevalence of each comorbidity. We analysed associations between T2DM and specific chronic diseases using logistic regression models.

**Results:**

More than 90% of elderly T2DM patients had at least one additional chronic disease. Multimorbidity was more frequent in women and older patients. Hyperlipidemia, hypertension, and ischaemic heart disease were the most prevalent comorbidities. The diagnosis of T2DM was significantly associated with both cardiovascular-kidney-metabolic and neuropsychiatric diseases. In addition, a higher prevalence and risk of chronic obstructive pulmonary disease (COPD) were consistently detected in elderly patients with T2DM, regardless of age and gender.

**Conclusions:**

Multimorbidity in elderly patients with T2DM needs broader acknowledgement. Current guidelines focus more on cardiovascular-kidney-metabolic and neuropsychiatric diseases with inadequate guidance on COPD management. Hence, the pleiotropic effects of glucose-lowering drugs on COPD should be further investigated to optimise the comprehensive management strategy for this population.

Type 2 diabetes mellitus (T2DM) increases the global economic burden and risk of premature death [1]. Widespread use of medications such as aspirin and angiotensin-converting enzyme inhibitors (ACEIs) has progressively reduced the affected population’s risk of cardiovascular death and increased their life expectancy, leading to a higher likelihood of other age-related chronic diseases [2]. Many studies have shown that most people with T2DM have other chronic conditions, with 40–50% having at least three [3,4]. This reflects the fundamental impact of long-term exposure to high glucose and insulin resistance on multiple organ systems, particularly the microvascular, macrovascular, and neuroimmune systems [5].

Multimorbidity, defined as the co-existence of two or more chronic conditions in an individual [6], leads to impaired physiological functioning [7], worsened frailty [8], and poorer quality of life [9], with increased risks of adverse outcomes, including polypharmacy [10], disability [11], and death [12], and a greater burden of disease [13]. A meta-analysis based on data from 193 studies conducted in various countries showed that the pooled prevalence of multimorbidity was 42.4% (95% confidence interval (CI) = 38.9–46.0%) [14]. Another recent systematic review showed that the prevalence of multimorbidity in Chinese adults was 25.4% (95% CI = 15.1–35.7), rising to 35.1% (95% CI = 23.9–46.3) in older adults (≥60 years), with geographically concentrated prevalence rates of over 50% in Fujian, Shanghai, and Hubei provinces [15]. Although T2DM is not the most common chronic disease among patients with multimorbidity, it has been shown that young patients with early-onset T2DM are at an increased risk of developing other comorbidities, particularly cardiovascular disease and end-stage renal disease, as a consequence of the prolonged duration of their T2DM [16–19]. To minimise the dramatic socioeconomic impact of T2DM, several researchers have already focussed on this young group [20,21]. However, health care systems have long been disease-centred rather than patient-centred. The complex health needs of elderly T2DM patients with multimorbidity present a challenge to health care providers and the health care system, both at the micro (ie, lifestyle interventions and therapeutic interventions) and macro (ie, coordination of care and system provision) levels [22].

In patients with T2DM, the nature of other comorbidities may influence the effectiveness of T2DM management [23]. Previous studies have primarily examined how specific or total counts of comorbidities affect T2DM management, with the concept of ‘concordant or discordant’ comorbidities offering insight into physician and patient priorities [2]. Concordant comorbidities refer to those conditions associated with T2DM in terms of pathogenesis or management plan, typically including cardiovascular-kidney-metabolic (CKM) diseases [24]. Concordant comorbidities, rather than discordant comorbidities, are the main focus of many specialist diabetes guidelines [20]. It has been demonstrated that the presence of concordant comorbidities (with or without discordant comorbidities) is associated with improved glycemic control in T2DM patients when compared to those with no comorbidities [26]. However, this correlation is not evident when only discordant comorbidities are present. Therefore, it is also important to understand the prevalence of discordant comorbidities and their impact on glycemic control in T2DM patients, such as neuropsychiatric diseases (NPDs) and respiratory diseases [25,26], to inform the subsequent development of evidence-based medical guidelines.

China has the world’s largest elderly population, with Shanghai having the highest proportion of older adults. We selected the Pudong New Area for its demographic similarity to the Shangai’s elderly population and its advanced foundation for health care data connectivity. The relatively high socioeconomic status of this region and large population of older T2DM patients increase the likelihood of a rise in multimorbid patients in the near future. We used electronic health data from primary care outpatient and all-level hospital inpatient settings in Pudong New Area to examine the prevalence of 12 common chronic diseases comorbid with T2DM in older adults during the 2019–22 period and further stratified the number of multimorbidity and prevalence of specific chronic diseases by age and gender. On this basis, we examined the prevalence trends of common comorbidities and assessed the impact of T2DM on the risk of comorbidities. This enabled us to evaluate whether these conditions are adequately addressed in Chinese diabetes guidelines for older adults.

## METHODS

### Data source and population

The database in this cross-sectional study came from the Shanghai Health Cloud platform, which aggregates health records from all public hospitals and community health centres in Shanghai, covering more than 99% of all residents. We used the unique identification codes to access the electronic health data of the residents of the Huamu community (Pudong New Area), including age, consultation time, and International Classification of Disease, 10th edition (ICD-10) codes diagnostic codes. We selected this community due to its geographic location, as it is situated in the central and western part of Pudong New Area, and the demographic structure of its population, which includes nearly 200 000 residents and almost 30 000 individuals over the age of 60 (following the World Health Organization's definition of older adults). Acquiring and accessing the above data required undergoing a vetting process and obtaining authorisation from the competent authorities.

We extracted the information on elderly (age ≥60 years) T2DM patients in the Huamu community during 2019–22 from the database. To obtain a medical diagnosis record for each patient, we included individuals with at least three documented outpatient visits or one documented hospitalisation each year. The index date was defined as the earliest date of T2DM diagnosis (ICD-10 codes: E11.2–E11.9). We calculated patients' age as their actual age on 31 December of each year. We excluded individuals whose age and sex could not be accurately identified. As the 12 common comorbidities are chronic and challenging to reverse in older patients, we reviewed electronic health records from 2018 to identify patients with missing T2DM diagnoses and comorbidities who did not attend a clinic visit in 2019. This approach was deemed necessary to ensure the accuracy and completeness of the T2DM and non-T2DM patient cohorts for subsequent analyses.

Furthermore, for each year of T2DM patients, we matched older patients without T2DM diagnosis 1:1 for age and gender to filter the control group for subsequent analyses.

### Measurement of multimorbidity

In this study, we defined multimorbidity as the co-existence of one or more other chronic diseases after the diagnosis of T2DM in T2DM patients or the co-existence of two or more other chronic diseases in non-T2DM patients. Considering the increased emphasis on standardised ICD-10 diagnostic codes at the primary care level in Shanghai around 2021, it is conceivable that some general practitioners may have been unable to employ the appropriate ICD-10 codes for the diagnosis of T2DM and comorbidities in their patients before this date. This has resulted in a relatively limited number of diagnoses that were not correctly coded. Accordingly, we employed an initial estimate to determine whether the proportion of any given ICD-10 code constituted less than 1% of the total number of diagnosis cases. Accordingly, a list of the most used ICD-10 codes for T2DM and potential comorbidities in the Huamu Community Health Service Centre was compiled and subsequently reviewed by trained physicians to identify patients' diagnoses in general hospitals. Following the recommendations of the international experts' consensus [27], chronic diseases, such as heart failure, peripheral arterial disease, asthma, Parkinson disease, epilepsy, dementia, schizophrenia, inflammatory bowel disease, and connective tissue diseases, should be included in the definition of multimorbidity. However, due to their relatively low proportion of diagnosed cases and the absence of their diagnosis and management in the routine work of primary care centres, we have excluded them from further consideration. Finally, we included the following chronic diseases: hypertension (HTN), hyperlipidaemia (HLP), ischaemic heart disease (IHD), stroke, chronic kidney disease (CKD), chronic obstructive pulmonary disease (COPD), non-alcoholic fatty liver disease (NAFLD), choleliths, cancer, insomnia, anxiety, and depression. Furthermore, we determined the diagnostic status of these comorbidities based on the time to diagnosis, the time to prescription, and/or the time to imaging.

Physicians then reviewed the list of ICD-10 codes for 10 common chronic diseases, excluding NAFLD and choleliths, which had been collated from online resources [28] and diagnostic records (Table S1 in the **Online Supplementary Document**). The list included the following codes: HTN (I10–I15), HLP (E78), IHD (I20–I25), stroke (I60, I61, I63 (except I63.6), I64, H34.1), CKD (N17–N19, N08.3, E11.2, E13.2, E14.2), COPD (J44), cancer (C00–C99), insomnia (G47), anxiety (F32.9), and depression (F41.1, R45.1). Following the methodology of our preceding study [29], clinical pharmacist reviewed pharmaceutical prescription records, while we calculated the cumulative defined daily doses based on the defined daily dose for each drug class. For pharmaceuticals with cumulative defined daily doses exceeding 180, they were aggregated according to the disease for which they were prescribed. This approach provided a more refined diagnosis of the diseases identified via ICD-10 codes. We identified NAFLD and choleliths manually using patients' abdominal ultrasound records, which ultimately provided all the information for older patients.

### Statistical analyses

First, based on the matched cross-sectional data set, we measured the proportion of different comorbidity numbers in T2DM patients and comparators annually during 2019–22 and we further calculated the mean number of comorbidities in each group stratified by both age (60–79, ≥80) and sex. Second, we estimated the annual prevalence rates and 95% CIs for each chronic disease in T2DM patients and comparators, as well as age/sex-specific annual prevalence rates. Similarly, we estimated the total and age/sex-specific annual prevalence rates for two categories of other CKM diseases (including HTN, HLP, IHD, stroke, and CKD) and NPDs, (including insomnia, anxiety, and depression) using the same methodology. We conducted logistic regression models to examine the associations between T2DM and each of the 12 conditions and to detect any differences in age and sex subgroups of these associations. Finally, we conducted sensitivity analyses in unmatched raw data sets.a

We performed all analyses using Stata, version 18.0 (StataCorp, Texas, US) and generated forest plots in GraphPad Prism, version 9.5.1 (GraphPad Software, California, US).

### Ethics

We conducted our study using retrospectively-obtained electronic health records following the approval of the Ethics Committee of Shanghai East Hospital (Approval No.2021YYS-203). We therefore did not require patient participation or informed consent.

## RESULTS

### Participants screening

**Figure 1:**
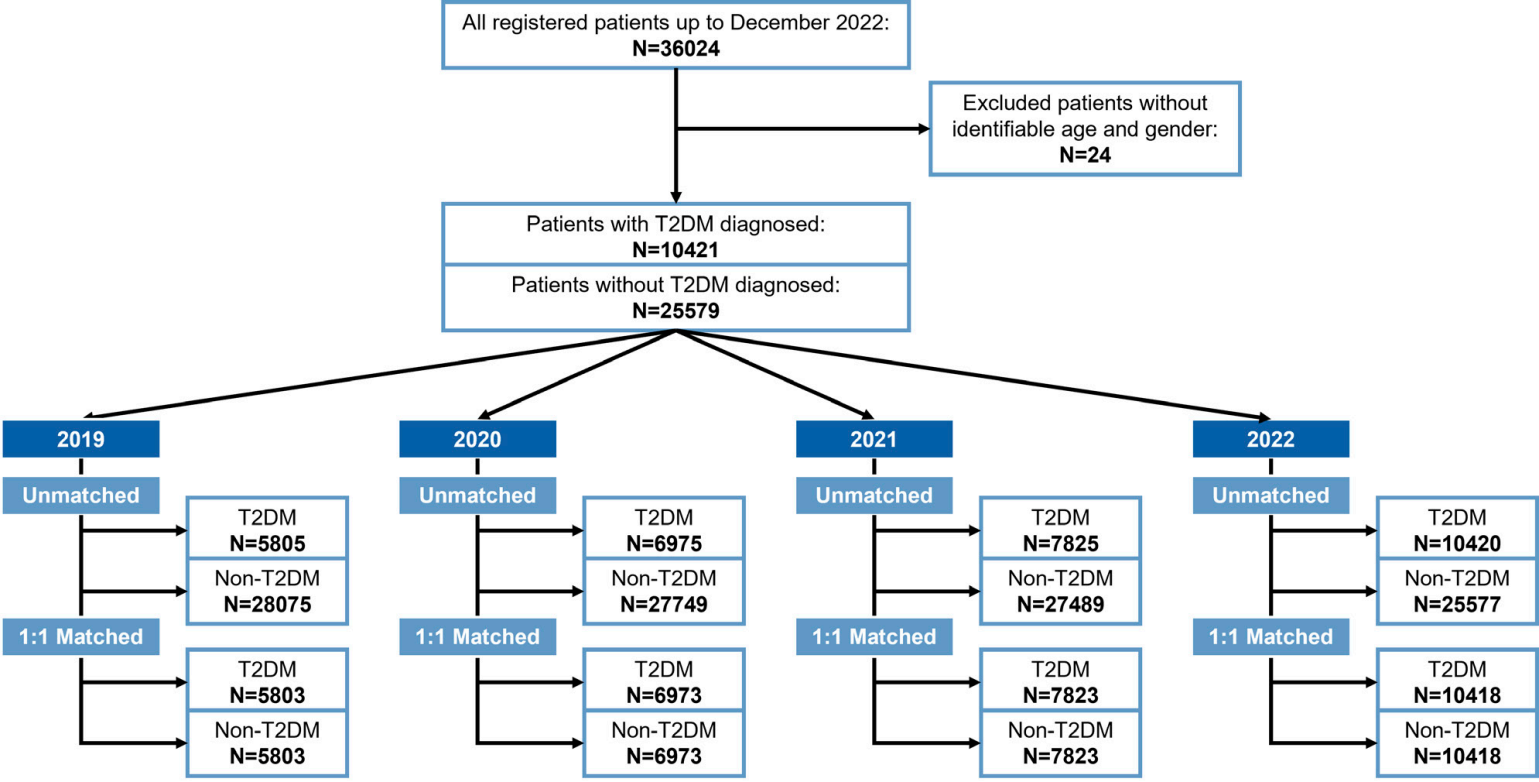
Flowchart for T2DM patients and comparators selected in each year. T2DM – type 2 diabetes mellitus.

We collected 36 024 patients' diagnosis and treatment records, through which we identified T2DM and non-T2DM patients each year according to the screening flowchart to construct annual cross-sections and perform matching (**Figure 1**).

### Proportion of multimorbidity

**Figure 2:**
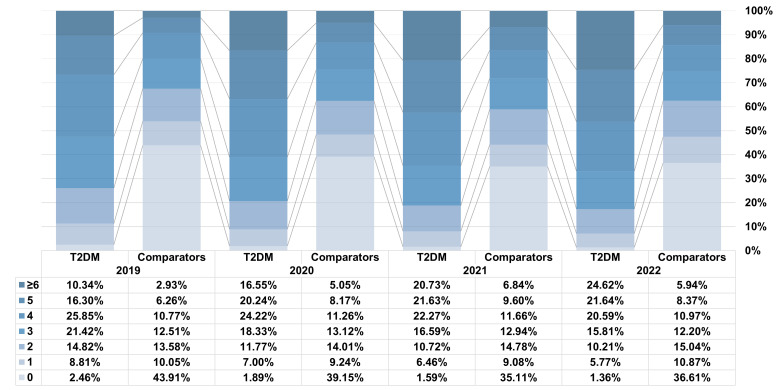
Proportion of people with T2DM and 1:1 matched comparators without T2DM by number of comorbidities, 2019–22. T2DM – type 2 diabetes mellitus.

**Figure 3:**
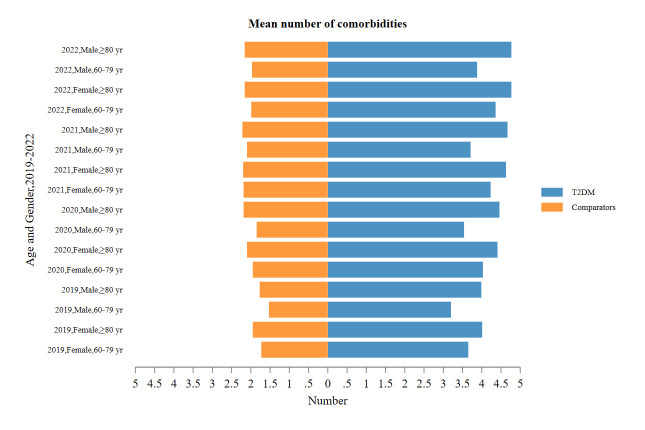
Mean number of comorbidities in T2DM cases and 1:1 matched comparators by age and gender, 2019–22. T2DM – type 2 diabetes mellitus.

All chronic diseases were more prevalent in T2DM patients than in matched comparators. A significantly higher proportion of T2DM patients presented with one or more comorbidities, collectively termed multimorbidity (**Figure 2**). In the year 2019, the prevalence of multimorbidity in T2DM patients was 97.54%, in comparison to a prevalence of 46% in comparators. Furthermore, the proportion of multimorbidity in T2DM patients increased to 98.67% in 2022, while the proportion of co-morbidities in comparators reached 52.52%. The proportions of T2DM patients and comparators in 2019 were 2.46% and 43.91% for those without any other chronic diseases, 8.81% and 10.05% for those with one comorbidity, 14.82% and 13.58% for those with two comorbidities, 21.42% and 12.51% for those with three comorbidities, 25.85% and 10.77% for those with four comorbidities, 16.30% and 6.26% for those with five comorbidities, and 10.34% and 2.93% for those with six or more comorbidities, respectively. The number of comorbidities tended to increase over time, both in matched cohorts and unmatched cohorts, especially in T2DM patients (Table S2 in the **Online Supplementary Document**). The mean numbers of comorbidities in T2DM patients were consistently more than twice as high as in comparators (**Figure 3**). When further grouped by age and sex, the number of comorbidities increased with age in T2DM patients and comparators, whether male or female; in the 60–79 age group, women had more comorbidities than men, both in T2DM patients and in comparators (Table S3 in the **Online Supplementary Document**).

### Annual prevalence rate of comorbidities

**Figure 4:**
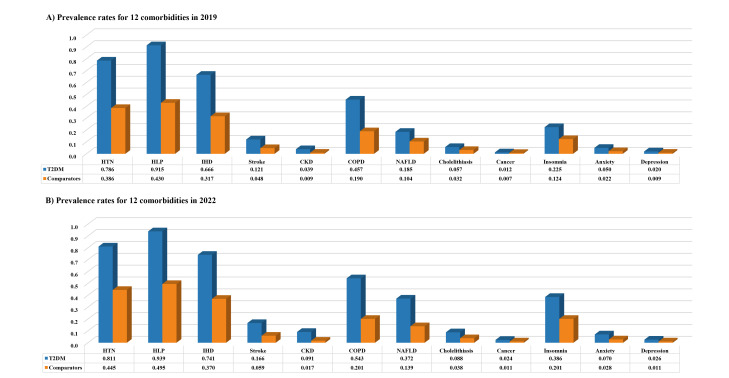
Annual prevalence rates for 12 comorbidities in T2DM cases and 1:1 matched comparators, 2019 vs. 2022. **Panel A.** Prevalence rates for 12 comorbidities in 2019. **Panel B.** Prevalence rates for 12 comorbidities in 2022. CKD – chronic kidney disease, COPD – chronic obstructive pulmonary disease, HLP – hyperlipidemia, HTN – hypertension, IHD – ischemic heart disease, NAFLD – non-alcoholic fatty liver disease, T2DM – type 2 diabetes mellitus.

All comorbidities were more prevalent in T2DM patients than comparators, with HLP, HTN, and IHD being the most predominant in all patients (Table S4 in the **Online Supplementary Document**). The prevalence of insomnia increased rapidly in both T2DM patients over the study period, from 22.5% (95% CI = 21.4–23.6) in 2019 to 38.6% (95% CI = 37.7–39.5) in 2022, as well as in comparators, from 12.4% (95% CI = 11.6–13.3) in 2019 to 20. 1% (95% CI = 19.4–20.9) in 2022. We only observed a rapid increase in NAFLD prevalence in T2DM patients, from 18.5% (95% CI = 17.5–19.5) in 2019 to 37.2% (95% CI = 36.3–38.2) in 2022 (**Figure 4**, Panels A–B). Cancer was the least common comorbidity in both groups.

We reported the age-specific and sex-specific prevalence of each comorbidity in T2DM patients and comparators in 2022 (Tables S5–6 in the **Online Supplementary Document**). When grouped by age, the prevalence of all major comorbidities except HTN and CKD increased with age in both T2DM patients and comparators (Figure S1, Panel A in the **Online Supplementary Document**). When grouped by sex, the prevalence of choleliths was higher in men than in women in T2DM patients, whereas the prevalence of HTN, CKD and cancer was higher in men than in women in the comparators, and other comorbidities were more common in women than in men. The prevalence of HLP, IHD, CKD, COPD, NAFLD, insomnia, anxiety, and depression was consistently higher in women than in men in both T2DM patients and comparators (Figure S1, Panel B in the **Online Supplementary Document**).

### Annual prevalence of CKM

The prevalence of CKM was consistently higher in T2DM patients than in matched comparators over the 2019–22 period, and the prevalence differences between these two groups remained similar when stratified by age and gender (Table S7 in the **Online Supplementary Document**). Whereas the prevalence of CKM in T2DM patients was higher in the group aged ≥80 or women than in the group aged 60–79 or men, the opposite trend was observed in comparators over time, with the prevalence of CKM lower in the group aged ≥80 or women than in the group aged 60–79 or men.

### Annual prevalence of NPD

The prevalence of NPDs was consistently higher in T2DM patients than in matched comparators, and the prevalence differences between these two groups were also consistent when stratified by age and gender (Table S8 in the **Online Supplementary Document**). In T2DM patients and comparators, NPD prevalence rates were higher in the group aged ≥80 or women than in the group aged 60–79 or men.

### Association of T2DM with comorbidities

**Figure 5:**
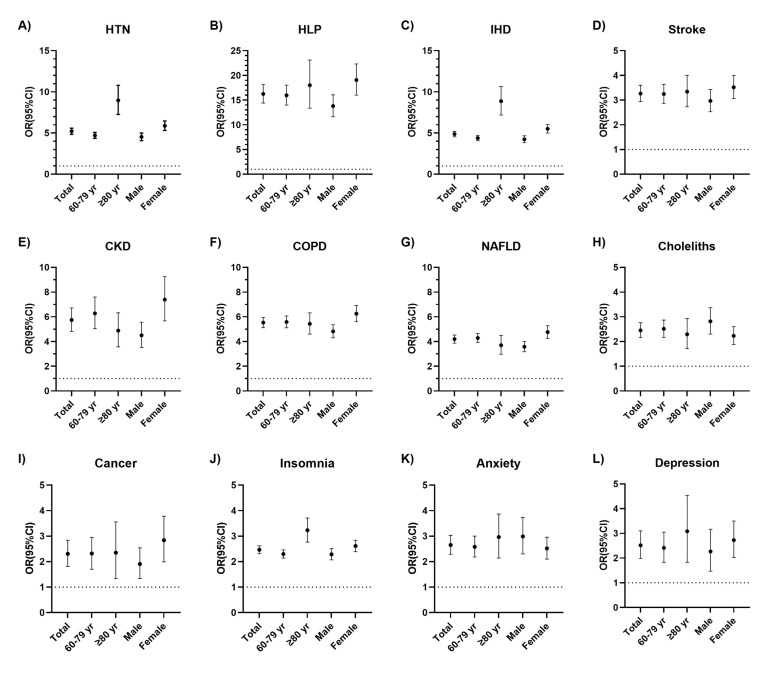
ORs (95% CI) for associations of T2DM with specific comorbidities in 2022 by conditional logistic regression models. **Panel A.** HTN. **Panel B.** HLP. **Panel C.** IHD. **Panel D.** Stroke. **Panel E.** CKD. **Panel F.** COPD. **Panel G.** NAFLD. **Panel H.** Choleliths. **Panel I.** Cancer. **Panel J.** Insomnia. **Panel K.** Anxiety. **Panel L.** Depression. CKD – chronic kidney disease, COPD – chronic obstructive pulmonary disease, HLP – hyperlipidemia, HTN – hypertension, IHD – ischemic heart disease, NAFLD – non-alcoholic fatty liver disease, T2DM – type 2 diabetes mellitus.

The diagnosis of T2DM was associated with a significantly increased risk of each comorbidity (**Figure 5**, Panels A–L). In 2022, T2DM patients were over three times more likely to have any CKM, including HTN (odds ratio (OR) = 5.21; 95% CI = 4.85–5.59), HLP (OR = 16.18; 95% CI = 14.41–18.16), IHD (OR = 4.87; 95% CI = 4.55–5.21), stroke (OR = 3.25; 95% CI = 2.9–3.60), and CKD (OR = 5.69; 95% CI = 4.82–6.72), than non-T2DM patients, while T2DM patients were about 2.5 times more likely to have any NPD, including insomnia (OR = 2.46; 95% CI = 2.31–2.62), anxiety (OR = 2.63; 95% CI = 2.29–3.03), and depression (OR = 2.48; 95% CI = 1.98–3.10) than non-T2DM patients. The ORs for all comorbidities except hypertension trended upwards during 2019–22 (Table S9 in the **Online Supplementary Document**).

T2DM diagnosis was associated with a significantly increased risk of each comorbidity among all age and gender subgroup. In age-specific analyses, older T2DM patients had higher risks of HTN, HLP, IHD, stroke, insomnia, anxiety, and depression, whereas T2DM patients aged 60–79 years had higher risks of CKD, COPD, NAFLD, choleliths, and cancer. In sex-specific analyses, women with T2DM had higher risks of all chronic conditions except choleliths and depression compared to men with T2DM.

We observed similar significant associations and results in general when using the original cross-sectional data sets for analyses (Table S10 in the **Online Supplementary Document**).

### Major comorbidity burden of T2DM

Irrespective of age and gender differences, the top four comorbidities in T2DM patients were HLP, HTN, IHD, and COPD, while the disease burden of NAFLD was more prominent in the younger elderly or female subgroups (Table S11 in the **Online Supplementary Document**). Among them, recommendations for COPD management were not included in the Guideline for the Management of Diabetes Mellitus in the Elderly in China [30].

## DISCUSSION

To our knowledge, this is one of the few real-world studies in China to investigate the prevalence of multimorbidity in elderly patients with T2DM. The results showed that comorbidities were more prevalent in elderly T2DM patients than in comparators during the study period of 2019–22, and that more than 97% of T2DM patients had at least one comorbidity defined as multimorbidity. We observed a higher prevalence of multimorbidity in women and patients aged ≥80 in this sample. This finding is in accordance with the results of the meta-analysis study [14], which indicated that the estimated prevalence of multimorbidity was higher in older patients. Another meta-analysis comprising 15.4 million individuals from 54 countries found the current global prevalence of multimorbidity to be 37.2% (95% CI = 34.9–39.4%), which was also more prevalent among older adults [31]. The two studies considered the possibility that records from primary care and health care institutions may result in a higher prevalence of multimorbidity. Both studies also corroborate our findings, where the prevalence of multimorbidity remained as high as 52.52% even in the control group. This can be attributed to a significant extent to changes in the disease spectrum resulting from the acceleration of the ageing process and the adoption of unhealthy lifestyles, in addition to other contributing factors.

In line with existing evidence [18,32,33], we saw a significant association between T2DM and other chronic diseases, particularly in the CKM and NPD categories. HLP, HTN and IHD were the most predominant comorbidities in elderly T2DM patients. These traditional comorbidities share complex direct or indirect effects [34] and common risk factors [35] with T2DM, such as unhealthy lifestyle, genetics and environmental factors, which may exacerbate the pathological process and disease progression. Besides, the high prevalence of NAFLD, another component of the metabolic syndrome, deserves more attention because of its relationship with insulin resistance and metabolic disorders in T2DM [36]. It is also conceivable that the extensive implementation of ultrasound diagnostics at the primary care level has resulted in the identification of numerous individuals with potentially undiagnosed NAFLD. As advocated by the American Heart Association [24], it is necessary to focus on the comprehensive management of CKM, especially in the high-risk group of older T2DM patients.

A did previous studies [32,37], we also investigated under-reported comorbidities in elderly T2DM patients, including COPD, anxiety and choleliths. The above non-traditional comorbidities are likely to share pathophysiological mechanisms such as chronic inflammation and oxidative stress with T2DM but tend to be underestimated in clinical guidelines for the general population. NPDs, such as depression and anxiety, can affect patients' ability to self-manage and thus impact long-term diabetes control and their risk of complications [38], while an unhealthy mood may also precipitate an increase in the prevalence of insomnia. However, compared with the previous version, the recommendations for comorbidity management proposed in the Chinese guideline for T2DM in the elderly emphasise CKM, NAFLD and NPD as priorities [30,39]. Therefore, future diabetes management strategies should consider a multidisciplinary collaborative model that includes mental health support and integrated CKM management.

Studies over the past decades have provided increasing evidence that the lung is a target organ for T2DM [40], most likely because of the systemic inflammatory response associated with T2DM patients [41,42]. There are similar inflammatory profiles between the two diseases, predominantly in neutrophils, macrophages and Th1 cells. Oxidative stress associated with high blood glucose levels may exacerbate bronchial inflammation, while exacerbation of the inflammatory response may further increase oxidative stress; this vicious cycle may then trigger lung function impairment, ultimately leading to a high prevalence of COPD in T2DM patients [40]. Despite this, a large randomised clinical trial demonstrated that the use of inhaled corticosteroid-long-acting beta-2 agonist combinations did not result in improved death or cardiovascular prognosis in COPD patients at high CVD risk, as a means of controlling airway inflammation [43]. Some studies have suggested that metformin may be important in inhibiting airway smooth muscle cell proliferation and reducing lung inflammation in T2DM patients with COPD [44]. This may potentially reduce the risk of COPD-related hospitalisations and all-cause mortality [45]. However, other studies have yielded conflicting results, which may be attributable to the fact that metformin reduces mitochondrial respiration in skeletal muscle as well as serum vitamin B12 levels [46–48]. Furthermore, there is evidence to suggest that mitochondrial dysfunction in skeletal muscle is associated with COPD disease severity, and lower serum vitamin B12 levels may have an impact on respiratory muscle function [46,49]. Thus, the pleiotropic effects of glucose-lowering drugs on COPD need to be investigated in more detail. For this reason, lifestyle interventions may represent the most fundamental and secure approach to health management at the primary care level for T2DM patients with comorbid COPD. Nevertheless, this remains a considerable challenge for patients with COPD [50]. To illustrate, in the case of interventions centred on physical activity, patients must cooperate with their general practicioners to surmount several hurdles, including, but not limited to, assisting them in effectively managing anxiety related to breathlessness [51].

Nevertheless, both Chinese and international guidelines on diabetes in the elderly contain fewer relative references to COPD [30,52]. As demonstrated by our analysis, the most significant comorbidities associated with T2DM are represented by the following conditions: HLP, HTN, and IHD. The reduction of cardiovascular mortality in patients through comprehensive lifestyle interventions and the early management of cardiometabolic risk has been recommended by experts, with the expectation that this approach will yield twice the results with half the effort [53]. In contrast, NAFLD, which has yet to be incorporated into the unified framework of CKMs, is strongly associated with the degree of obesity among T2DM patients [54]. Moreover, prolonged fatty infiltration results in liver fibrosis, which can contribute to an elevated risk of cardiovascular disease [55,56] and chronic kidney disease [57,58] in patients with T2DM. As previously discussed, the management of these comorbidities can often be effectively integrated to reduce the self-management burden on patients. Nevertheless, further clinical research is required, conducted by both specialists and general practicioners, to promote the development of high-quality evidence-based guidelines. This is particularly the case in the areas of barriers and facilitation strategies relating to lifestyle interventions [51], as well as how to identify potentially inappropriate medication use and to avoid prescribing cascades due to polypharmacy [50], for patients with COPD who have not been prioritised and included in guidelines. Considering the growing prominence of the biopsychosocial model of medicine, it is becoming increasingly evident that T2DM guidelines are shifting their attention towards NPDs, such as insomnia, anxiety, and depression.

Additionally, age and gender have a significant effect on the spectrum of multimorbidity in T2DM patients. In this study, we found that the number of comorbidities in female T2DM patients was generally higher than in males, especially in the 60–79 age group. As with previous meta-analytic findings [31], even when not accounting for a T2DM diagnosis, the prevalence of multimorbidity is significantly higher in women (39.4%; 95% CI = 36.4–42.4) compared to men (32.8%; 95% CI = 30.0–35.6%). This phenomenon may be related to changes in hormone levels, physiological structural differences and increased susceptibility to certain diseases in post-menopausal women [50]. Prior research [32,59] has indicated that women may be more inclined to seek primary care than men. This has resulted in a higher prevalence of comorbidities being identified and subsequently diagnosed in women with T2DM. Older age is another important predictor of multimorbidity, mainly due to the physiological decline and chronic inflammation in older people, which increases their susceptibility to more chronic diseases [15], especially in T2DM patients. Although the impact of multimorbidity on the risk of adverse outcomes in older T2DM patients is well known, including increased health care utilisations and risk of disability and death, the relationship between multimorbidity and glucose control (mainly Hb1Ac) remains to be better elucidated [60,61].

A major strength of our study is the integration of primary and secondary care records and self-reported data from community-dwelling elderly patients, supplemented by prescription data, which allowed complete and accurate prevalence estimates for each chronic condition, with particularly high sensitivity for identifying patients with T2DM. Second, the longer study period ensured the statistical validity of the prevalence trends for the major chronic conditions, as well as the total, sex and age-specific prevalence estimates, and guided the optimisation of subsequent community-based T2DM management strategies. In addition, to avoid the influence of confounders, we performed conditional logistic regression analyses using a 1:1 matching approach and discussed differences in the association of T2DM diagnosis with each major chronic condition across age and sex subgroups. Finally, our findings focussed on non-traditional comorbidities that are relevant to patients' quality of life, in addition to these 'traditional' comorbidities, and also examined how well they were covered in national guidelines for the management of older people with T2DM.

Unfortunately, there are some limitations to this study. Due to unsatisfactory consistency in the quality of diagnostic and treatment data among different medical institutions in different regions of China, we could not directly conduct a nationwide multicentre study and only used single-central data from a representative community in Pudong New Area. It remains possible that patients with undiagnosed conditions may have been misclassified, for example, some of the patients with abnormal fasting glucose were not further diagnosed as having T2DM and were instead classified into comparators. Similarly, the diagnosis of NAFLD and cholelithiasis based on abdominal ultrasound imaging is dependent on the subjective interpretation of physicians, which can result in false-negative outcomes. The elimination of misclassification is also a challenging process due to the presence of inadequate or erroneous recording. The potential for such discrepancies is reduced by the quality control measures that are employed during the linking process, which occurs prior to the data collection phase [62]. Alternatively, CKD screening in primary care has been gradually expanded in China in recent years, so the increase in CKD prevalence observed needs to be treated with caution, and there may still be a large number of undiagnosed or undertreated CKD patients. Second, the comorbidity categories were not exhaustive and did not include gastrointestinal diseases, dementia, arthritis, etc. This was mainly because there were fewer records of the above conditions in the main index data (primary care outpatient records), and the lack of standardised diagnostic and treatment capacity of general practitioners made it difficult to accurately estimate the exact prevalence of these conditions. It is therefore imperative to consider the inclusion of as many comorbidities as possible in the event that data accessibility improves in the future. Therefore, we are currently engaged in discussions with relevant organisations with a view to establishing a reliable database of primary care research in our region, with reference to the Clinical Practice Research Datalink [63]. Furthermore, it is crucial to explore the complex associations and intrinsic mechanisms between T2DM and comorbidities by means of techniques such as latent category analysis and network analysis. Finally, discussing the pooled effect of lifestyle on multimorbidity is precluded by missing data in the administrative data set. The limitations and the characteristics of cross-sectional analyses preclude the accurate inference of causality between T2DM and comorbidities, even with the application of more robust statistical methods. Consequently, this study is unable to draw such conclusions.

A further objective is to conduct a longitudinal cohort study of community-based elderly patients. This will facilitate the identification of more accurate patterns of multimorbidity and enable the monitoring of individuals' health status over time using AI and big data technologies. This approach will elucidate the dynamic associations between patient phenotypes, inter-individual overlapping characteristics and the risk of health outcomes. By engaging in a coordinated collaboration between medical experts, data scientists and artificial intelligence/information technology researchers [64], an interdisciplinary knowledge base will be constructed, which will inform the development of novel digital health management guidelines in the future.

## CONCLUSIONS

Multimorbidity in patients with T2DM warrants greater attention and individualised patient-centred management. In our study, the prevalence of comorbidities in T2DM patients was much higher than in comparators and increased with age. Except for cholelithiasis, other comorbidities were more prevalent in women. The management of COPD in T2DM patients has not yet been specifically addressed in guidelines, compared with CKM and NPD. The pleiotropic effects of glucose-lowering drugs on COPD should be further investigated to provide a precise integrated management strategy, based on comprehensive cardiometabolic management and psychosocial support.

## Additional material


Online Supplementary Document
