# Supplementary material for: Multimorbidity in elderly patients with or without T2DM: A real-world cross-sectional analysis based on primary care and hospitalisation data
Source: J Glob Health. 2024 Dec 20;14:04263. doi: 10.7189/jogh.14.04263 (PMC11658714; doi:10.7189/jogh.14.04263)
Supplement: Online Supplementary Document [file jogh-14-04263-s001.pdf]

Table S1 ICD-10 codes for identifying 10 comorbidities (except NAFLD and choleliths)

**Disease**	**ICD-10 codes**
Anxiety	F32.9
Cancer	C00-C99
Chronic kidney disease	N17-N19, N08.3, E11.2, E13.2, E14.2
Chronic obstructive pulmonary disease	J44
Depression	F41.1, R45.1
Hyperlipidemia	E78
Hypertension	I10-I15
Insomnia	G47
Ischemic heart disease	I20-I25
Stroke	I60, I61, I63 (except I63.6), I64, H34.1

Table S2 Number of comorbidities in T2DM cases and comparators with or without matched, 2019-2022

**Number of comorbidities**		**2019**		**2020**		**2021**		**2022**	
		**T2DM**	**Comparators**	**T2DM**	**Comparators**	**T2DM**	**Comparators**	**T2DM**	**Comparators**
0	Unmatched	143(2.46%)	12741(45.38%)	132(1.89%)	11014(39.69%)	124(1.58%)	9751(35.47%)	142(1.36%)	9254(36.18%)
	**Matched**	**143(2.46%)**	**2548(43.91%)**	**132(1.89%)**	**2730(39.15%)**	**124(1.59%)**	**2747(35.11%)**	**142(1.36%)**	**3814(36.61%)**
1	Unmatched	512(8.81%)	2774(9.88%)	488(7.00%)	2652(9.565)	505(6.45%)	2548(9.27%)	601(5.77%)	2720(10.63%)
	**Matched**	**511(8.81%)**	**583(10.05%)**	**488(7.00%)**	**644(9.24%)**	**505(6.46%)**	**710(9.08%)**	**601(5.77%)**	**1132(10.87%)**
2	Unmatched	861(14.83%)	3902(13.90%)	822(11.78%)	3948(14.23%)	840(10.73%)	3978(14.47%)	1065(10.22%)	3987(15.59%)
	**Matched**	**860(14.82%)**	**788(13.58%)**	**821(11.77%)**	**977(14.01%)**	**839(10.72%)**	**1156(14.78%)**	**1064(10.21%)**	**1567(15.04%)**
3	Unmatched	1243(21.41%)	3465(12.34%)	1278(18.32%)	3474(12.52%)	1298(16.59%)	3599(13.09%)	1647(15.81%)	3250(12.71%)
	**Matched**	**1243(21.42%)**	**726(12.51%)**	**1278(18.33%)**	**915(13.12%)**	**1298(16.59%)**	**1012(12.94%)**	**1647(15.81%)**	**1271(12.20%)**
4	Unmatched	1500(25.84%)	2859(10.18%)	1689(24.22%)	3181(11.46%)	1742(22.26%)	3300(12.00%)	2145(20.59%)	2796(10.93%)
	**Matched**	**1500(25.85%)**	**625(10.77%)**	**1689(24.22%)**	**785(11.26%)**	**1742(22.27%)**	**912(11.66%)**	**2145(20.59%)**	**1143(10.97%)**
5	Unmatched	946(16.30%)	1608(5.73%)	1411(20.23%)	2196(7.91%)	1693(21.64%)	2555(9.29%)	2254(21.63%)	2128(8.32%)
	**Matched**	**946(16.30%)**	**363(6.26%)**	**1411(20.24%)**	**570(8.17%)**	**1693(21.64%)**	**751(9.60%)**	**2254(21.64%)**	**872(8.37%)**
⩾6	Unmatched	600(10.34%)	726(2.59%)	1155(19.90%)	1284(4.63%)	1623(20.74%)	1758(6.40%)	2566(24.63%)	1442(5.64%)
	**Matched**	**600(10.34%)**	**170(2.93%)**	**1154(16.55%)**	**352(5.05%)**	**1622(20.73%)**	**535(6.84%)**	**2565(24.62%)**	**619(5.94%)**

Table S3 Mean number of comorbidities in T2DM and comparators by age and gender, 2019-2022

	**T2DM**				**Comparators**			
	**Male**		**Female**		**Male**		**Female**	
	**60-79 yr**	**⩾80 yr**	**60-79 yr**	**≥80 yr**	**60-79 yr**	**⩾80 yr**	**60-79 yr**	**⩾80 yr**
2019	3.20(1.56)	3.99(1.46)	3.65(1.61)	4.01(1.51)	1.53(1.73)	1.77(2.01)	1.73(1.89)	1.95(2.03)
2020	3.54(1.64)	4.46(1.57)	4.03(1.72)	4.41(1.59)	1.85(1.89)	2.19(2.22)	1.95(1.99)	2.10(2.12)
2021	3.71(1.70)	4.67(1.60)	4.23(1.81)	4.63(1.62)	2.10(1.96)	2.22(2.26)	2.19(2.06)	2.20(2.33)
2022	3.88(1.76)	4.77(1.63)	4.36(1.82)	4.77(1.67)	1.97(1.91)	2.16(2.25)	1.99(2.00)	2.16(2.23)

Table S4 Annual prevalence rates for 12 comorbidities in T2DM cases and comparators with or without matched, 2019-2022

**Comorbidity**		**2019**		**2020**		**2021**		**2022**	
		**T2DM**	**Comparators**	**T2DM**	**Comparators**	**T2DM**	**Comparators**	**T2DM**	**Comparators**
HTN	Unmatched	0.786(0.775,0.796)	0.377(0.371,0.383)	0.807(0.797,0.816)	0.423(0.417,0.429)	0.814(0.806,0.823)	0.459(0.453,0.465)	0.811(0.804,0.819)	0.449(0.443,0.455)
	**Matched**	**0.786(0.775,0.796)**	**0.386(0.373,0.398)**	**0.807(0.797,0.816)**	**0.428(0.418,0.440)**	**0.814(0.805,0.823)**	**0.464(0.453,0.475)**	**0.811(0.804,0.819)**	**0.445(0.435,0.454)**
HLP	Unmatched	0.914(0.907,0.921)	0.418(0.412,0.424)	0.934(0.928,0.940)	0.486(0.480,0.492)	0.942(0.937,0.948)	0.534(0.528,0.540)	0.939(0.935,0.944)	0.506(0.500,0.512)
	**Matched**	**0.915(0.907,0.922)**	**0.430(0.417,0.443)**	**0.934(0.928,0.940)**	**0.493(0.481,0.504)**	**0.942(0.937,0.948)**	**0.541(0.530,0.552)**	**0.939(0.935,0.944)**	**0.495(0.485,0.505)**
IHD	Unmatched	0.665(0.653,0.677)	0.304(0.299,0.310)	0.715(0.704,0.726)	0.356(0.350,0.361)	0.746(0.736,0.755)	0.395(0.389,0.401)	0.741(0.732,0.749)	0.373(0.367,0.379)
	**Matched**	**0.666(0.653,0.678)**	**0.317(0.305,0.329)**	**0.715(0.704,0.726)**	**0.361(0.350,0.373)**	**0.746(0.736,0.755)**	**0.402(0.391,0.413)**	**0.741(0.732,0.749)**	**0.370(0.361,0.379)**
Stroke	Unmatched	0.121(0.113,0.130)	0.040(0.037,0.042)	0.149(0.141,0.158)	0.054(0.051,0.056)	0.162(0.154,0.171)	0.063(0.060,0.066)	0.166(0.159,0.173)	0.058(0.055,0.061)
	**Matched**	**0.121(0.113,0.130)**	**0.048(0.042,0.054)**	**0.149(0.141,0.158)**	**0.058(0.052,0.064)**	**0.162(0.154,0.171)**	**0.066(0.060,0.071)**	**0.166(0.159,0.173)**	**0.059(0.054,0.063)**
CKD	Unmatched	0.039(0.034,0.044)	0.007(0.006,0.008)	0.066(0.060,0.072)	0.013(0.011,0.014)	0.083(0.077,0.089)	0.017(0.015,0.018)	0.091(0.085,0.096)	0.018(0.017,0.020)
	**Matched**	**0.039(0.034,0.044)**	**0.009(0.007,0.012)**	**0.065(0.060,0.071)**	**0.015(0.013,0.019)**	**0.083(0.077,0.089)**	**0.018(0.015,0.021)**	**0.091(0.085,0.096)**	**0.017(0.015,0.020)**
COPD	Unmatched	0.457(0.444,0.470)	0.179(0.175,0.184)	0.523(0.511,0.534)	0.204(0.200,0.209)	0.536(0.525,0.547)	0.216(0.211,0.221)	0.543(0.533,0.552)	0.194(0.190,0.199)
	**Matched**	**0.457(0.444,0.470)**	**0.190(0.180,0.200)**	**0.523(0.511,0.534)**	**0.214(0.204,0.223)**	**0.536(0.525,0.547)**	**0.214(0.205,0.224)**	**0.543(0.533,0.552)**	**0.201(0.194,0.209)**
NAFLD	Unmatched	0.185(0.175,0.195)	0.099(0.096,0.103)	0.234(0.224,0.244)	0.122(0.118,0.126)	0.267(0.257,0.277)	0.148(0.143,0.152)	0.372(0.363,0.381)	0.129(0.125,0.134)
	**Matched**	**0.185(0.175,0.195)**	**0.104(0.097,0.112)**	**0.234(0.224,0.244)**	**0.125(0.117,0.133)**	**0.267(0.257,0.277)**	**0.149(0.141,0.157)**	**0.372(0.363,0.382)**	**0.139(0.132,0.145)**
Choleliths	Unmatched	0.057(0.051,0.063)	0.030(0.028,0.032)	0.067(0.061,0.073)	0.036(0.034,0.038)	0.069(0.063,0.075)	0.040(0.037,0.042)	0.088(0.082,0.093)	0.036(0.033,0.038)
	**Matched**	**0.057(0.051,0.063)**	**0.032(0.027,0.037)**	**0.067(0.061,0.073)**	**0.037 (0.033,0.042)**	**0.069(0.064,0.075)**	**0.038(0.034,0.042)**	**0.088(0.082,0.093)**	**0.038(0.035,0.042)**
Cancer	Unmatched	0.012(0.009,0.015)	0.006(0.005,0.007)	0.016(0.014,0.020)	0.009(0.008,0.010)	0.020(0.017,0.024)	0.010(0.009,0.011)	0.024(0.021,0.027)	0.010(0.009,0.011)
	**Matched**	**0.012(0.009,0.015)**	**0.007(0.005,0.009)**	**0.016(0.014,0.020)**	**0.010(0.007,0.012)**	**0.020(0.017,0.024)**	**0.010(0.008,0.013)**	**0.024(0.021,0.027)**	**0.011(0.009,0.013)**
Insomnia	Unmatched	0.225(0.214,0.236)	0.116(0.113,0.120)	0.314(0.303,0.325)	0.169(0.164,0.173)	0.374(0.363,0.385)	0.209(0.205,0.214)	0.386(0.377,0.396)	0.203(0.198,0.208)
	**Matched**	**0.225(0.214,0.236)**	**0.124(0.116,0.133)**	**0.314(0.303,0.325)**	**0.172(0.163,0.181)**	**0.374(0.363,0.385)**	**0.213(0.204,0.222)**	**0.386(0.377,0.395)**	**0.201(0.194,0.209)**
Anxiety	Unmatched	0.050(0.044,0.056)	0.018(0.017,0.020)	0.060(0.054,0.066)	0.024(0.022,0.026)	0.069(0.063,0.075)	0.029(0.027,0.031)	0.070(0.065,0.075)	0.027(0.026,0.030)
	**Matched**	**0.050(0.044,0.056)**	**0.022(0.018,0.026)**	**0.060(0.054,0.066)**	**0.023(0.020,0.027)**	**0.069(0.063,0.075)**	**0.031(0.027,0.035)**	**0.070(0.065,0.075)**	**0.028(0.025,0.031)**
Depression	Unmatched	0.020(0.017,0.024)	0.008(0.007,0.009)	0.024(0.020,0.027)	0.010(0.009,0.011)	0.026(0.022,0.029)	0.011(0.010,0.012)	0.026(0.023,0.029)	0.010(0.009,0.012)
	**Matched**	**0.020(0.017,0.024)**	**0.009(0.007,0.012)**	**0.024(0.020,0.027)**	**0.010(0.008,0.013)**	**0.026(0.022,0.029)**	**0.013(0.011,0.016)**	**0.026(0.023,0.029)**	**0.011(0.009,0.013)**

* Abbreviations: HTN, Hypertension; HLP, Hyperlipidemia; IHD, Ischemic heart disease; CKD, Chronic kidney disease; COPD, Chronic obstructive pulmonary disease; NAFLD, Non-alcoholic fatty liver disease.

Table S5 Age-specific prevalence rates for 12 comorbidities in T2DM cases and 1:1 matched comparators, 2019 vs 2022

	**HTN**	**HLP**	**IHD**	**Stroke**	**CKD**	**COPD**	**NAFLD**	**Choleliths**	**Cancer**	**Insomnia**	**Anxiety**	**Depression**
2019												
60-79 yr												
T2DM	0.766	0.908	0.639	0.106	0.034	0.440	0.200	0.056	0.013	0.204	0.049	0.019
Comparators	0.377	0.427	0.305	0.042	0.008	0.182	0.112	0.032	0.007	0.115	0.021	0.08
≥80 yr												
T2DM	0.881	0.944	0.793	0.197	0.062	0.540	0.108	0.063	0.006	0.328	0.052	0.025
Comparators	0.428	0.446	0.376	0.075	0.013	0.230	0.064	0.030	0.007	0.168	0.025	0.013
2022												
60-79 yr												
T2DM	0.792	0.937	0.713	0.146	0.078	0.513	0.402	0.087	0.024	0.360	0.065	0.024
Comparators	0.442	0.497	0.359	0.051	0.014	0.187	0.151	0.037	0.011	0.195	0.027	0.010
≥80 yr												
T2DM	0.896	0.949	0.863	0.255	0.143	0.675	0.243	0.091	0.026	0.501	0.089	0.037
Comparators	0.453	0.486	0.419	0.091	0.033	0.265	0.085	0.042	0.012	0.229	0.032	0.013

* Abbreviations: HTN, Hypertension; HLP, Hyperlipidemia; IHD, Ischemic heart disease; CKD, Chronic kidney disease; COPD, Chronic obstructive pulmonary disease; NAFLD, Non-alcoholic fatty liver disease.

Table S6 Gender-specific prevalence rates for 12 comorbidities in T2DM cases and 1:1 matched comparators, 2019 vs 2022

	**HTN**	**HLP**	**IHD**	**Stroke**	**CKD**	**COPD**	**NAFLD**	**Choleliths**	**Cancer**	**Insomnia**	**Anxiety**	**Depression**
2019												
Male												
T2DM	0.763	0.905	0.610	0.105	0.040	0.415	0.165	0.056	0.011	0.204	0.035	0.011
Comparators	0.388	0.416	0.282	0.046	0.007	0.167	0.099	0.030	0.006	0.110	0.015	0.005
Female												
T2DM	0.806	0.923	0.714	0.136	0.038	0.494	0.202	0.058	0.012	0.243	0.062	0.028
Comparators	0.383	0.442	0.347	0.049	0.011	0.210	0.109	0.033	0.008	0.137	0.028	0.012
2022												
Male												
T2DM	0.800	0.932	0.697	0.150	0.087	0.512	0.332	0.089	0.023	0.346	0.056	0.018
Comparators	0.471	0.502	0.355	0.057	0.021	0.198	0.135	0.034	0.013	0.186	0.020	0.008
Female												
T2DM	0.821	0.945	0.777	0.179	0.094	0.568	0.405	0.087	0.025	0.419	0.081	0.033
Comparators	0.423	0.489	0.383	0.059	0.014	0.204	0.141	0.042	0.009	0.214	0.034	0.013

* Abbreviations: HTN, Hypertension; HLP, Hyperlipidemia; IHD, Ischemic heart disease; CKD, Chronic kidney disease; COPD, Chronic obstructive pulmonary disease; NAFLD, Non-alcoholic fatty liver disease.

Table S7 Annual prevalence rates for cardiovascular-kidney-metabolic diseases in T2DM cases and 1:1 matched comparators, 2019-2022

	**Total**	**Age-spesific**		**Gender-specific**	
		**60-79 yr**	**⩾80 yr**	**Male**	**Female**
2019					
T2DM	0.962	0.956	0.987	0.953	0.969
Comparators	0.507	0.501	0.535	0.491	0.521
2020					
T2DM	0.971	0.968	0.987	0.964	0.977
Comparators	0.552	0.551	0.559	0.560	0.555
2021					
T2DM	0.975	0.972	0.990	0.969	0.980
Comparators	0.599	0.610	0.551	0.602	0.596
2022					
T2DM	0.972	0.969	0.978	0.965	0.978
Comparators	0.569	0.574	0.549	0.579	0.562

Table S8 Annual prevalence rates for neuropsychiatric diseases in T2DM cases and 1:1 matched comparators, 2019-2022

	**Total**	**Age-spesific**		**Gender-specific**	
		**60-79 yr**	**⩾80 yr**	**Male**	**Female**
2019					
T2DM	0.398	0.374	0.518	0.352	0.439
Comparators	0.232	0.223	0.278	0.207	0.254
2020					
T2DM	0.399	0.372	0.521	0.356	0.438
Comparators	0.224	0.219	0.250	0.205	0.242
2021					
T2DM	0.395	0.366	0.514	0.349	0.435
Comparators	0.226	0.222	0.244	0.211	0.240
2022					
T2DM	0.394	0.370	0.502	0.351	0.430
Comparators	0.205	0.198	0.237	0.188	0.219

TableS9 Odds ratios (95% CI) for associations of T2DM with specific comorbidities in annual matched cross-sections 2019-2022 by conditional logistic regression models

	**HTN**	**HLP**	**IHD**	**Stroke**	**CKD**	**COPD**	**NAFLD**	**Choleliths**	**Cancer**	**Insomnia**	**Anxiety**	**Depression**
2019												
Total	5.62 (5.11,6.18)	15.13 (13.10,17.46)	4.54 (4.15,4.98)	2.88 (2.47,3.35)	4.35 (3.22,5.88)	3.83 (3.49,4.21)	2.02 (1.80,2.25)	1.84 (1.53,2.21)	1.74 (1.17,2.59)	2.09 (1.88,2.31)	2.35 (1.90,2.90)	2.27 (1.64,3.16)
60-79 yr	5.21 (4.71,5.77)	13.88 (11.93,16.15)	4.27 (3.87,4.71)	2.77 (2.33,3.29)	4.23 (2.98,6.00)	3.82 (3.44,4.24)	2.05 (1.82,2.31)	1.78 (1.46,2.18)	1.94 (1.25,2.99)	1.99 (1.77,2.23)	2.41 (1.90,3.06)	2.39 (1.64,3.50)
⩾80 yr	8.72 (6.65,11.45)	26.89 (17.01,42.52)	6.28 (4.95,7.98)	3.22 (2.37,4.37)	4.69 (2.58,8.54)	3.89 (3.14,4.81)	1.79 (1.29,2.48)	2.10 (1.35,3.27)	0.86 (.029,2.55)	2.46 (1.97,3.08)	2.08 (1.28,3.39)	1.92 (0.98,3.76)
Male	4.89 (4.28,5.58)	14.30 (11.68,17.51)	4.10 (3.60,4.66)	2.53 (2.02,3.18)	5.45 (3.38,8.78)	3.79 (3.29,4.37)	1.87 (1.58,2.21)	1.90 (1.44,2.50)	1.93 (1.04,3.61)	2.13 (1.82,2.50)	2.47 (1.70,3.61)	2.14 (1.14,4.04)
Female	6.41 (5.60,7.34)	15.96 (13.03,19.55)	5.00 (4.40,5.68)	3.17 (2.59,3.88)	3.66 (2.47,5.41)	3.86 (3.40,4.38)	2.14 (1.84,2.48)	1.79 (1.39,2.29)	1.61 (0.96,2.71)	2.05 (1.79,2.35)	2.29 (1.77,2.96)	2.32 (1.58,3.42)
2020												
Total	5.41 (4.95,5.90)	16.09 (13.97,19.53)	4.57 (4.21,4.97)	2.92 (2.57,3.30)	4.45 (3.58,5.52)	4.54 (4.16,4.95)	2.26 (2.06,2.49)	1.88 (1.60,2.20)	1.73 (1.28,2.34)	2.23 (2.06,2.43)	2.68 (2.22,3.23)	2.42 (1.82,3.21)
60-79 yr	4.86 (4.43,5.33)	14.93 (12.85,17.36)	4.21 (3.85,4.60)	2.91 (2.52,3.36)	4.93 (3.79,6.40)	4.41 (4.00,4.86)	2.26 (2.04,2.51)	1.76 (1.48,2.09)	1.92 (1.37,2.70)	2.05 (1.86,2.25)	2.78 (2.25,3.43)	2.22 (1.61,3.06)
⩾80 yr	10.37 (7.94,13.55)	25.17 (16.59,38.19)	7.32 (5.80,9.23)	2.93 (2.30,3.74)	3.50 (2.39,5.12)	5.09 (4.17,6.21)	2.28 (1.74,3.00)	2.61 (1.74,3.89)	1.07 (0.53,2.16)	3.06 (2.54,3.68)	2.36 (1.60,3.49)	3.23 (1.73,6.02)
Male	4.49 (3.98,5.07)	13.67 (11.30,16.55)	3.88 (3.47,4.35)	2.21 (1.85,2.64)	4.04 (2.97,5.49)	4.17 (3.67,4.74)	2.11 (1.83,2.44)	2.12 (1.67,2.70)	1.12 (.074,1.68)	2.14 (1.88,2.42)	2.31 (1.70,3.13)	1.72 (1.05,2.82)
Female	6.46 (5.69,7.33)	19.09 (15.46,23.57)	5.38 (4.77,6.06)	3.73 (3.13,4.46)	4.86 (3.58,6.59)	4.87 (4.32,5.50)	2.39 (2.10,2.72)	1.70 (1.38,2.11)	2.87 (1.79,4.61)	2.32 (2.07,2.59)	2.91 (2.30,3.69)	2.83 (1.99,4.03)
2021												
Total	4.97 (4.58,5.39)	14.42 (12.63,16.47)	4.43 (4.10,4.79)	2.84 (2.54,3.18)	5.17 (4.26,6.28)	5.12 (4.69,5.58)	2.23 (2.05,2.43)	1.90 (1.64,2.20)	1.98 (1.51,2.58)	2.23 (2.07,2.40)	2.31 (1.98,2.70)	1.98 (1.56,2.52)
60-79 yr	4.31 (3.95,4.70)	13.50 (11.67,15.61)	3.92 (3.60,4.26)	2.77 (2.43,3.16)	6.01 (4.70,7.69)	5.02 (4.55,5.54)	2.25 (2.05,2.47)	1.87 (1.58,2.20)	2.13 (1.57,2.90)	1.99 (1.84,2.16)	2.29 (1.91,2.73)	1.91 (1.44,2.52)
⩾80 yr	11.35 (8.81,14.63)	19.05 (13.81,26.29)	8.66 (6.912,10.84)	3.03 (2.45,3.74)	3.88 (2.82,5.32)	5.46 (4.54,6.57)	2.13 (1.70,2.66)	2.04 (1.47,2.82)	1.50 (0.85,2.64)	3.39 (2.85,4.02)	2.39 (1.73,3.32)	2.19 (1.39,3.45)
Male	4.09 (3.66,4.58)	14.17 (11.67,17.21)	4.07 (3.64,4.55)	2.52 (2.14,2.98)	4.60 (3.49,6.05)	4.79 (4.21,5.45)	1.98 (1.74,2.25)	2.21 (1.76,2.76)	1.51 (1.02,2.24)	2.02 (1.81,2.25)	2.14 (1.66,2.75)	2.07 (1.33,3.22)
Female	6.03 (5.34,6.79)	14.64 (12.21,17.54)	4.78 (4.29,5.33)	3.12 (2.68,3.64)	5.77 (4.38,7.59)	5.40 (4.80,6.07)	2.45 (2.18,2.76)	1.69 (1.39,2.05)	2.46 (1.70,3.57)	2.41 (2.18,2.66)	2.42 (1.98,2.95)	1.94 (1.46,2.58)

2022												
Total	5.21<br>(4.85,5.59)	16.18<br>(14.41,18.16)	4.87<br>(4.55,5.21)	3.25<br>(2.94,3.60)	5.69<br>(4.82,6.72)	5.53<br>(5.12,5.96)	4.19<br>(3.87,4.53)	2.44<br>(2.16,2.76)	2.27<br>(1.81,2.84)	2.46<br>(2.31,2.62)	2.63<br>(2.29,3.03)	2.48<br>(1.98,3.10)
60-79 yr	4.71<br>(4.36,5.08)	15.88<br>(13.98,18.04)	4.38<br>(4.08,4.72)	3.23<br>(2.87,3.64)	6.19<br>(5.04,7.60)	5.57<br>(5.11,6.07)	4.28<br>(3.93,4.66)	2.50<br>(2.17,2.87)	2.30<br>(1.78,2.95)	2.29<br>(2.14,2.46)	2.56<br>(2.18,3.00)	2.36<br>(1.83,3.05)
⩾80 yr	8.85<br>(7.26,10.79)	17.57<br>(13.36,23.12)	8.75<br>(7.19,10.65)	3.30<br>(2.73,4.00)	4.75<br>(3.57,6.33)	5.39<br>(4.60,6.32)	3.65<br>(2.97,4.49)	2.24<br>(1.71,2.93)	2.17<br>(1.33,3.56)	3.21<br>(2.77,3.71)	2.88<br>(2.14,3.87)	2.88<br>(1.83,4.54)
Male	4.51<br>(4.07,5.00)	13.68<br>(11.64,16.07)	4.23<br>(3.84,4.67)	2.95<br>(2.53,3.43)	4.42<br>(3.52,5.56)	4.80<br>(4.30,5.36)	3.56<br>(3.17,4.01)	2.78<br>(2.30,3.37)	1.84<br>(1.34,2.54)	2.28<br>(2.07,2.51)	2.93<br>(2.30,3.74)	2.16<br>(1.47,3.17)
Female	5.86<br>(5.32,6.47)	18.90<br>(16.00,22.32)	5.50<br>(5.00,6.05)	3.50<br>(3.06,4.00)	7.25<br>(5.67,9.26)	6.23<br>(5.61,6.93)	4.74<br>(4.26,5.28)	2.21<br>(1.88,2.60)	2.75<br>(1.99,3.78)	2.61<br>(2.39,2.84)	2.49<br>(2.10,2.96)	2.66<br>(2.02,3.50)

* Abbreviations: HTN, Hypertension; HLP, Hyperlipidemia; IHD, Ischemic heart disease; CKD, Chronic kidney disease; COPD, Chronic obstructive pulmonary disease; NAFLD, Non-alcoholic fatty liver disease.

Table S10 Odds ratios (95% CI) for associations of T2DM with specific comorbidities in annual unmatched cross-sections 2019-2022 by logistic regression models

	HTN	HLP	IHD	Stroke	CKD	COPD	NAFLD	Choleliths	Cancer	Insomnia	Anxiety	Depression
2019												
Total	6.00 (5.61,6.42)	14.84 (13.50,16.32)	4.55 (4.29,4.84)	3.26 (2.95,3.60)	5.33 (4.40,6.45)	3.82 (3.60,4.06)	2.05 (1.90,2.21)	1.90 (1.67,2.17)	1.94 (1.46,2.58)	2.16 (2.01,2.32)	2.80 (2.41,3.25)	2.43 (1.94,3.05)
60-79 yr	5.47 (5.09,5.88)	14.09 (12.73,15.59)	4.30 (4.02,4.59)	3.11 (2.77,3.49)	5.03 (4.02,6.29)	3.68 (3.44,3.94)	1.96 (1.81,2.13)	1.76 (1.52,2.03)	1.98 (1.46,2.68)	2.08 (1.92,2.26)	3.00 (2.54,3.54)	2.38 (1.85,3.07)
⩾80 yr	10.37 (8.47,12.69)	20.79 (15.76,27.41)	6.06 (5.13,7.16)	3.19 (2.62,3.89)	5.21 (3.57,7.62)	4.11 (3.55,4.75)	1.97 (1.54,2.51)	1.95 (1.43,2.66)	1.03 (0.42,2.53)	2.48 (2.12,2.90)	1.93 (1.37,2.71)	2.37 (1.45,3.89)
Male	5.12 (4.66,5.63)	13.81 (12.08,15.77)	4.10 (3.76,4.47)	2.78 (2.38,3.24)	5.11 (3.87,6.75)	3.55 (3.24,3.89)	1.86 (1.66,2.10)	2.06 (1.69,2.50)	1.67 (1.09,2.56)	2.13 (1.91,2.38)	2.58 (2.00,3.33)	1.85 (1.21,2.84)
Female	6.96 (6.33,7.65)	15.94 (13.92,18.26)	5.04 (4.63,5.49)	3.67 (3.21,4.18)	5.51 (4.22,7.18)	4.06 (3.74,4.40)	2.20 (1.99,2.44)	1.79 (1.50,2.13)	2.19 (1.49,3.22)	2.19 (1.99,2.40)	2.91 (2.43,3.49)	2.72 (2.09,3.55)
2020												
Total	5.66 (5.31,6.03)	15.13 (13.73,16.68)	4.56 (4.31,4.83)	3.05 (2.80,3.32)	5.39 (4.67,6.21)	4.30 (4.06,4.55)	2.19 (2.05,2.33)	1.88 (1.68,2.11)	1.86 (1.48,2.32)	2.24 (2.11,2.38)	2.60 (2.29,2.95)	2.42 (1.99,2.95)
60-79 yr	5.11 (4.77,5.48)	14.53 (13.08,16.15)	4.19 (3.93,4.46)	2.99 (2.71,3.30)	5.24 (4.43,6.19)	4.09 (3.84,4.35)	2.12 (1.97,2.28)	1.72 (1.52,1.96)	1.88 (1.48,2.40)	2.09 (1.95,2.32)	2.61 (2.26,3.00)	2.24 (1.79,2.81)
⩾80 yr	9.96 (8.27,11.99)	19.92 (15.38,25.79)	7.46 (6.34,8.80)	2.84 (2.41,3.35)	4.95 (3.75,6.53)	5.17 (4.52,5.91)	2.18 (1.78,2.65)	2.05 (1.57,2.68)	1.23 (0.69,2.20)	3.07 (2.69,3.51)	2.42 (1.84,3.19)	2.92 (1.94,4.40)
Male	4.80 (4.39,5.26)	13.59 (11.86,15.56)	3.97 (3.66,4.31)	2.58 (2.26,2.93)	4.95 (4.02,6.10)	3.97 (3.66,4.32)	1.99 (1.80,2.20)	2.10 (1.77,2.49)	1.51 (1.08,2.11)	2.19 (2.00,2.40)	2.30 (1.86,2.85)	1.72 (1.19,2.47)
Female	6.59 (6.02,7.22)	16.83 (14.63,19.37)	5.24 (4.82,5.69)	3.46 (3.09,3.88)	5.78 (4.76,7.03)	4.60 (4.27,4.97)	2.35 (2.15,2.57)	1.72 (1.47,2.00)	2.22 (1.64,3.00)	2.29 (2.11,2.48)	2.77 (2.37,3.24)	2.82 (2.24,3.57)
2021												
Total	5.15 (4.85,5.48)	14.66 (13.28,16.17)	4.51 (4.26,4.77)	2.88 (2.67,3.12)	5.29 (4.68,5.98)	4.32 (4.09,4.56)	2.10 (1.98,2.23)	1.78 (1.60,1.98)	2.01 (1.65,2.44)	2.26 (2.14,2.39)	2.50 (2.24,2.80)	2.41 (2.01,2.89)
60-79 yr	4.55 (4.25,4.86)	14.07 (12.64,15.67)	4.07 (3.83,4.33)	2.79 (2.54,3.06)	5.32 (4.59,6.18)	4.18 (3.93,4.44)	2.05 (1.91,2.19)	1.64 (1.46,1.85)	1.99 (1.60,2.48)	2.07 (1.95,2.21)	2.47 (2.17,2.81)	2.20 (1.78,2.72)
⩾80 yr	10.07 (8.45,11.99)	20.10 (15.71,25.72)	8.00 (6.83,9.36)	2.85 (2.46,3.30)	4.55 (3.65,5.67)	5.07 (4.48,5.74)	2.29 (1.93,2.71)	1.93 (1.52,2.45)	1.65 (1.06,2.59)	3.26 (2.89,3.68)	2.55 (2.02,3.22)	2.92 (2.04,4.18)
Male	4.26 (3.90,4.65)	12.97 (11.30,14.87)	3.93 (3.63,4.26)	2.57 (2.29,2.90)	4.80 (4.01,5.76)	3.99 (3.68,4.32)	1.89 (1.72,2.07)	1.97 (1.68,2.31)	1.49 (1.10,2.01)	2.11 (1.94,2.29)	2.28 (1.89,2.75)	2.05 (1.48,2.83)
Female	6.15 (5.63,6.71)	16.51 (14.34,19.02)	5.18 (4.78,5.62)	3.14 (2.83,3.49)	5.73 (4.85,6.78)	4.64 (4.31,4.99)	2.29 (2.12,2.48)	1.64 (1.42,1.89)	2.57 (1.98,3.35)	2.39 (2.22,2.57)	2.64 (2.29,3.04)	2.60 (2.09,3.24)

2022												
Total	5.28 (5.00,5.58)	15.43 (14.18,16.79)	4.84 (4.60,5.09)	3.34 (3.10,3.60)	5.52 (4.93,6.19)	5.28 (5.02,5.56)	3.99 (378,4.21)	2.60 (2.37,2.86)	2.48 (2.08,2.95)	2.48 (2.36,2.61)	2.66 (2.39,2.96)	2.59 (2.18,3.08)
60-79 yr	4.76 (4.49,5.05)	16.36 (14.90,17.96)	4.44 (4.20,4.69)	3.32 (3.04,3.64)	5.68 (4.92,6.55)	5.08 (4.79,5.38)	3.98 (3.75,4.23)	2.45 (2.20,2.73)	2.37 (1.94,2.90)	2.35 (2.22,2.48)	2.63 (2.32,2.97)	2.40 (1.96,2.93)
⩾80 yr	10.11 (8.65,11.82)	19.11 (15.49,23.57)	8.32 (7.22,9.59)	2.95 (2.57,3.38)	4.32 (3.56,5.25)	5.47 (4.88,6.13)	3.31 (2.86,3.83)	2.27 (1.84,2.80)	2.03 (1.39,2.96)	3.38 (3.03,3.78)	2.64 (2.13,3.28)	2.91 (2.07,4.08)
Male	4.46 (4.11,4.83)	13.36 (11.85,15.07)	4.11 (3.82,4.42)	2.91 (2.60,3.26)	4.83 (4.09,5.71)	4.66 (4.32,5.02)	3.40 (3.13,3.69)	2.79 (2.42,3.22)	2.02 (1.57,2.61)	2.29 (2.12,2.47)	2.67 (2.24,3.19)	2.50 (1.84,3.40)
Female	6.12 (5.67,6.60)	17.59 (15.62,19.81)	5.61 (5.22,6.02)	3.69 (3.34,4.07)	6.15 (5.26,7.19)	5.84 (5.45,6.26)	4.51 (4.19,4.84)	2.46 (2.16,2.79)	2.96 (2.32,3.80)	2.63 (2.46,2.81)	2.64 (2.31,3.02)	2.62 (2.13,3.23)

Abbreviations: HTN, Hypertension; HLP, Hyperlipidemia; IHD, Ischemic heart disease; CKD, Chronic kidney disease; COPD, Chronic obstructive pulmonary disease; NAFLD, Non-alcoholic fatty liver disease.

Table S11 Major comorbidities in elderly patients with T2DM, 2022

	**Comorbidities with prevalence⩾30% in T2DM cases**	**Comorbidities with OR ⩾4 refered to comparators**	**Comorbidities with prevalence⩾30% and OR ⩾4**
Total	**1. HLP(93.9%)**	**1. HLP(16.18)**	**1. HLP(93.9%,OR=16.18)**
	2. HTN(81.1%)	2. CKD(5.69)	2. HTN(81.1%,OR=5.21)
	3. IHD(74.1%)	3. COPD(5.53)	3. IHD(74.1%,OR=4.87)
	4. COPD(54.3%)	4. HTN(5.21)	4. COPD(54.3%,OR=5.53)
	5. Insomnia(38.6%)	5. IHD(4.87)	5. NAFLD(37.2%,OR=4.19)
	6. NAFLD(37.2%)	6. NAFLD(4.19)	
60-79 yr	**1. HLP(93.7%)**	**1. HLP(15.88)**	**1. HLP(93.7%,OR=15.88)**
	2. HTN(79.2%)	2. CKD(6.19)	2. HTN(79.2%,OR=4.71)
	3. IHD(71.3%)	3. COPD(5.57)	3. IHD(71.3%,OR=4.38)
	4. COPD(51.3%)	4. HTN(4.71)	4. COPD(51.3%,OR=5.57)
	5. NAFLD(40.2%)	5. IHD(4.38)	5. NAFLD(40.2%,OR=4.28)
	6. Insomnia(36.0%)	6. NAFLD(4.28)	
⩾80 yr	**1. HLP(94.9%)**	**1. HLP(17.57)**	**1. HLP(94.9%,OR=17.57)**
	2. HTN(89.6%)	2. HTN(8.85)	2. HTN(89.6%,OR=8.85)
	3. IHD(89.6%)	3. IHD(8.75)	3. IHD(89.6%,OR=8.75)
	4. COPD(67.5%)	4. COPD(5.39)	4. COPD(67.5%,OR=5.39)
	5. Insomnia(50.1%)	5. CKD(4.75)	
Male	**1. HLP(93.2%)**	**1. HLP(13.68)**	**1. HLP(93.2%,OR=13.68)**
	2. HTN(80.0%)	2. COPD(4.80)	2. HTN(80.0%,OR=4.51)
	3. IHD(69.7%)	3. HTN(4.51)	3. IHD(69.7%,OR=4.23)
	4. COPD(51.2%)	4. CKD(4.42)	4. COPD(51.2%,OR=4.80)
	5. Insomnia(34.6%)	5. IHD(4.23)	
	6. NAFLD(33.2%)		
Female	**1. HLP(94.5%)**	**1. HLP(18.90)**	**1. HLP(94.5%,OR=18.90)**
	2. HTN(82.1%)	2. CKD(7.25)	2. HTN(82.1%,OR=5.86)
	3. IHD(77.7%)	3. COPD(6.23)	3. IHD(77.7%,OR=5.60)
	4. COPD(56.8%)	4. HTN(5.86)	4. COPD(56.8%,OR=6.23)
	5. Insomnia(41.9%)	5. IHD(5.60)	5. NAFLD(40.5%,OR=4.74)
	6. NAFLD(40.5%)	6. NAFLD(4.74)	

Abbreviations: HTN, Hypertension; HLP, Hyperlipidemia; IHD, Ischemic heart disease; COPD, Chronic obstructive pulmonary disease; NAFLD, Non-alcoholic fatty liver disease.

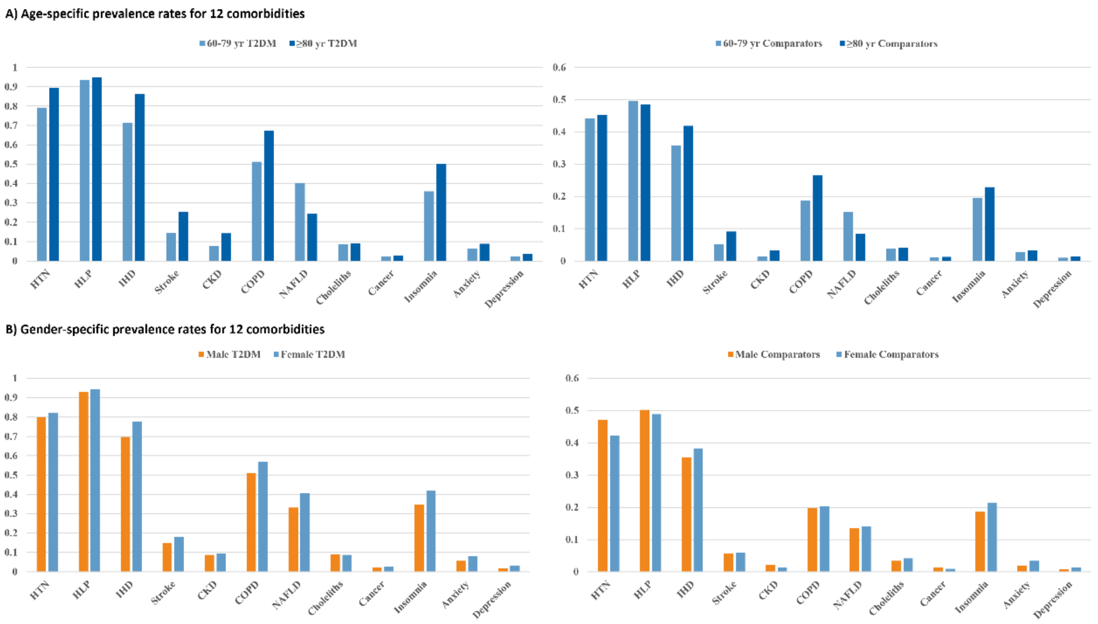

Figure S1 Age-specific and gender-specific prevalence rates for 12 comorbidities in patients with type 2 diabetes (T2DM) and matched comparators without diabetes, 2022. HTN, Hypertension; HLP, Hyperlipidemia; IHD, Ischemic heart disease; CKD, Chronic kidney disease; COPD, Chronic obstructive pulmonary disease; NAFLD, Non-alcoholic fatty liver disease.
